# Exosome-mediated communication between gastric cancer cells and macrophages: implications for tumor microenvironment

**DOI:** 10.3389/fimmu.2024.1327281

**Published:** 2024-02-22

**Authors:** Yue Qiu, Guimei Lu, Na Li, Yanyan Hu, Hao Tan, Chengyao Jiang

**Affiliations:** ^1^ Medical Oncology Department of Gastrointestinal Cancer, Cancer Hospital of China Medical University, Liaoning Cancer Hospital and Institute, Shenyang, Liaoning, China; ^2^ Department of Laboratory, Cancer Hospital of China Medical University, Liaoning Cancer Hospital and Institute, Shenyang, Liaoning, China; ^3^ Thoracic Esophageal Radiotherapy Department, Cancer Hospital of China Medical University, Liaoning Cancer Hospital and Institute, Shenyang, Liaoning, China; ^4^ Department of Gastric Surgery, Cancer Hospital of China Medical University, Liaoning Cancer Hospital & Institute, Shenyang, Liaoning, China

**Keywords:** gastric cancer (GC), tumor-associated macrophages (TAMs), exosome, tumor microenvironment (TME), immune

## Abstract

Gastric cancer (GC) is a malignant neoplasm originating from the epithelial cells of the gastric mucosa. The pathogenesis of GC is intricately linked to the tumor microenvironment within which the cancer cells reside. Tumor-associated macrophages (TAMs) primarily differentiate from peripheral blood monocytes and can be broadly categorized into M1 and M2 subtypes. M2-type TAMs have been shown to promote tumor growth, tissue remodeling, and angiogenesis. Furthermore, they can actively suppress acquired immunity, leading to a poorer prognosis and reduced tolerance to chemotherapy. Exosomes, which contain a myriad of biologically active molecules including lipids, proteins, mRNA, and noncoding RNAs, have emerged as key mediators of communication between tumor cells and TAMs. The exchange of these molecules via exosomes can markedly influence the tumor microenvironment and consequently impact tumor progression. Recent studies have elucidated a correlation between TAMs and various clinicopathological parameters of GC, such as tumor size, differentiation, infiltration depth, lymph node metastasis, and TNM staging, highlighting the pivotal role of TAMs in GC development and metastasis. In this review, we aim to comprehensively examine the bidirectional communication between GC cells and TAMs, the implications of alterations in the tumor microenvironment on immune escape, invasion, and metastasis in GC, targeted therapeutic approaches for GC, and the efficacy of potential GC drug resistance strategies.

## 1 Introduction

GC is a malignant tumor originating from the epithelium of the gastric mucosa, which can be detected in any part of the stomach, but commonly in the antrum, greater curvature, lesser curvature, and anterior and posterior wall of the stomach (1, 2). The causative ingredients of GC are too varied to be fully identified. Among different elements, Helicobacter pylori (Hp) infection is thought to be a significant pathogenic key player for GC (3, 4). In the diagnosis of GC, the early symptoms of GC are not clear; only symptoms such as epigastric discomfort bear a strong resemblance between diverse chronic diseases like gastritis and gastric ulcer without specificity, leading to a very low detection proportion of early GC (5, 6), which is as high as 90%, so more specific and effective ways of GC diagnosis are necessary. Nowadays, the therapy for GC is mainly surgical, combined with perioperative neoadjuvant chemotherapy, adjuvant chemotherapy, radiotherapy, targeted therapy, and other comprehensive means (7, 8). However, the initial manifestation of GC often involves subtle clinical symptoms, which, combined with the absence of distinctive diagnostic indicators, implies that a considerable number of patients have already advanced to intermediate and advanced stages of the disease before being diagnosed. Consequently, the prognosis for these patients remains particularly unfavorable, with a low 5-year survival rate. While the meaning of the paragraph remains the same, the revised version incorporates more formal language and restructures the sentences for improved logic and flow (9, 10). Thus, it is extremely significant to investigate the pathogenesis of GC and seek diagnostic markers with high specificity for the diagnosis and treatment of GC.

The tumor microenvironment (TME) consists of physical, biochemical, and cellular components that are linked with biological processes such as tumor development and tumor immune escape (11, 12). The function of immune cells in the tumor microenvironment and tumor malignant progression is of great importance (13, 14). Different types of cytokines can be produced by immune cells as a means of exerting negative regulation on immune function and promoting tumor growth. Among these, tumor-associated macrophages (TAMs) are the most abundant (15, 16). Macrophages in the normal body have valued biological functions, including processing and presenting antigens, regulating immunity, resisting microorganisms, clearing foreign bodies, phagocytosis, and tissue remodeling (17–20). In specific microenvironments, macrophages polarize into subtypes with various biological capabilities, i.e., M1-type macrophages and M2-type macrophages (21–23). Inflammatory responses are a prominent part of the microenvironment of GC immunity. TAMs, among the inflammatory cells involved in the response, have a prominent role in enhancing the development of GC. Investigations have found that a large number of various immune cells can infiltrate both primary and metastatic tumors, in which the number of TAMs tends to have a momentous predominance (24, 25). The majority of the TAMs have an M2-type phenotype with a weak antigen-presenting capacity, whose antitumor influences are inhibited, benefiting tumor growth and metastasis (26). Despite the fact that the tumor-promoting mechanism of TAMs is not well understood, the number of TAMs is usually negatively connected with the prognosis of tumor patients.

Exosomes are lipid microvesicles of endocytotic origin (27, 28), which play a crucial role in various diseases like cardiovascular diseases, hematologic diseases, autoimmune diseases, and tumors (29–32). Exosomes are present in almost all living cells and can exist stably in diverse body fluids, like saliva, plasma, milk, urine, cerebrospinal fluid, bile, etc. (33–35). Exosome cytoplasm involves microRNA (miRNA), long noncoding RNA (lncRNA), messenger RNA (mRNA), proteins, lipids, and lots of other active ingredients (36–38). Exosomes may deliver the active ingredients, which they convey to the target cells through mutual fusion with the cell membranes of the recipient cells, cytosis, and other modes of action to impact the function of recipient cells (39–41).

To sum up, this paper offers an overview of the intercommunication between GC cells and TAMs and the influence of alterations in the tumor microenvironment on GC immune escape, invasion, and metastasis, GC-targeted therapeutic strategies, and the effect on GC drug resistance.

## 2 The overview of exosomes

**Figure 1:**
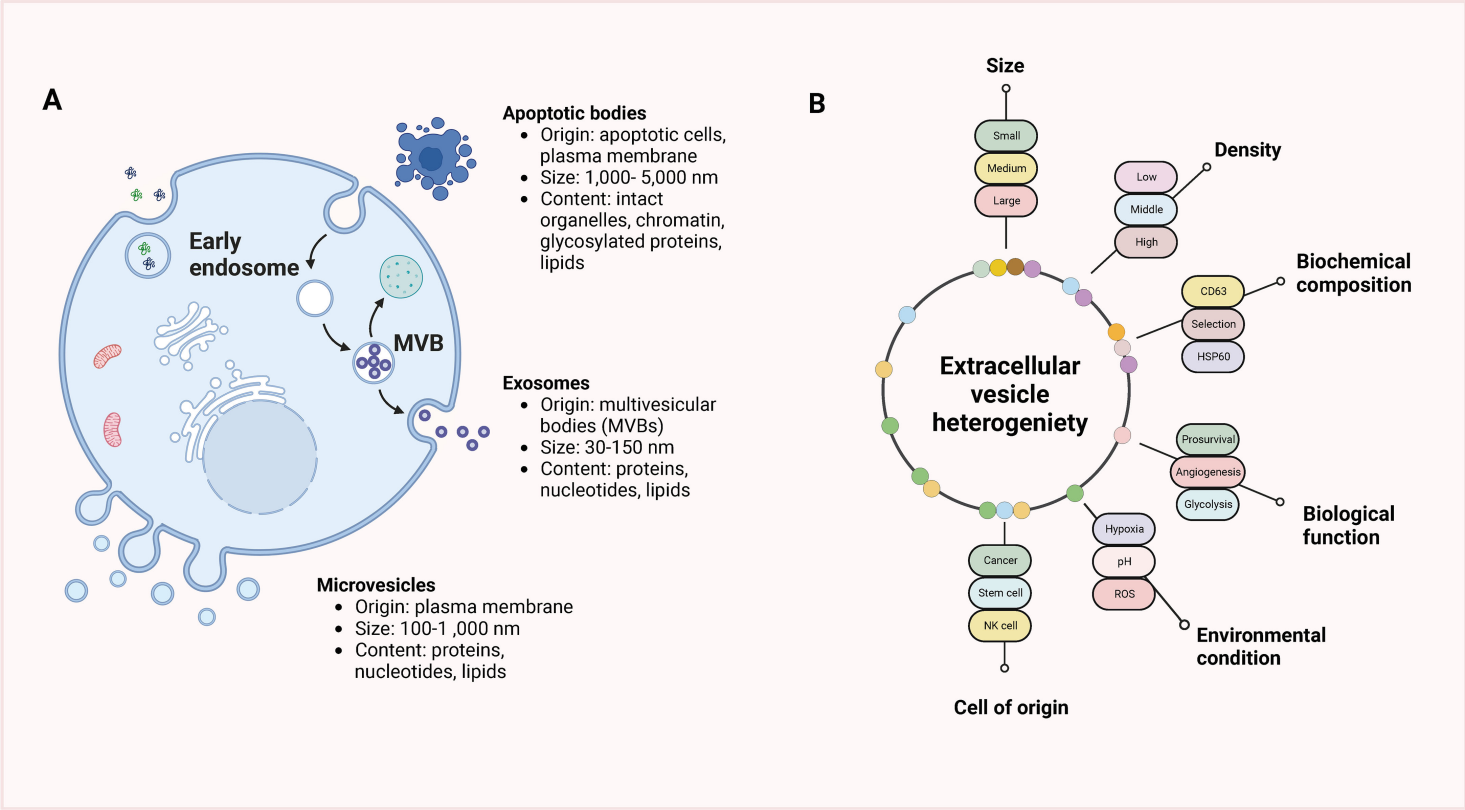
Classification of extracellular vesicles and classification criteria. **(A)** EVs are categorized into three main subtypes based on their origin, size, and content: apoptotic bodies, microvesicles, and exosomes. Apoptotic bodies are larger vesicles released from dying cells during the process of programmed cell death (apoptosis). Microvesicles, also known as ectosomes, are formed by outward budding and shedding of the plasma membrane and are typically larger than exosomes. Exosomes, on the other hand, are small vesicles of endocytic origin, originating from the inward budding of endosomal membranes. **(B)** The heterogeneity of EVs presents various criteria that can be used for their classification. EVs can be classified based on factors such as size, density, biochemical composition, biological function, environmental conditions, or the cell of origin. These classification criteria help in distinguishing and characterizing different types of EVs, which can vary in terms of their cargo, signaling molecules, and functions.

Extracellular vesicles (EVs) can carry substances such as proteins, lipids, and nucleic acids and are structures enclosed by unit membranes secreted by cells under physiological or pathological conditions. According to the different secretion methods, extracellular vesicles can be divided into microvesicles, exosomes, apoptotic bodies, and migratory bodies. The common classification of extracellular vesicles and classification criteria are shown in **Figure 1**. Cells secrete exosomes with a particle size of typically 30–150 nm, which are membranous extracellular vesicles formed prominently through endosomal membrane invaginations (42–46). Exosomes consist of diverse crucial biomolecules, such as the four transmembrane protein family (CD9, CD63, CD81), growth factors (TGFB 1, bFGF), major histocompatibility complexes (MHC I/MHcII), adhesion proteins, heat shock proteins, enzymes, lipids, nucleic acids, and a small portion of genetic material, of which CD9, CD63, CD81, HSP70Alix, etc. have been considered specific marker proteins in exosome isolation and identification (47–49). During the formation of vesicles, numerous biomolecules in the cytoplasm are also encapsulated in the vesicles. It is a significant reason why exosomes can reflect the cellular status and disease development process (50–52). Research has shown that there are varying physiological functions exosomes have that are derive from dissimilar tissues and cellular sources. Exosome-mediated intercellular communication is not only involved in the regulation of normal physiological processes but also in the pathological processes of different diseases, including cancer, which has resulted in a great abundance of interest in the investigation of tumor diagnosis and treatment (53–56). Scientists found that TAMs can also exchange information with tumor cells through exosomes and are instrumental in a variety of tumor processes (57–59).

Since exosomes change morphology, purity, and biological activity during isolation and preparation, the characterization of exosomes becomes one of the key and most difficult issues in new research (60, 61). Scientists have applied numerous modern techniques to the characterization of exosomes, including transmission electron microscopy, dynamic light scattering, nano-tracking analysis techniques, protein concentration assays, protein blotting, and flow cytometry (62–65). Transmission electron microscopy is a kind of microscope that uses an electron beam to evince the surface or interior of a sample, which allows the observation of the size and morphology of exosomes without specific proteins on the surface of the exosomes. Dynamic light scattering can detect the particle size distribution of exosomes but is sensitive to external conditions. The nanoparticle tracking analysis technique is an approach that applies light scattering and Brownian motion to count exosomes, but it cannot analyze the morphology and characterize certain proteins of exosomes. Protein blotting and flow cytometric analysis can take advantage of the principle of antigen–antibody binding to analyze specific proteins on the surface of exosomes, but the particle count cannot be known. As a result, the present study and development of the components and properties of exosomes still have a huge amount of space. More modern and advanced technologies are necessary for their characterization and analysis.

## 3 The overview of TAMs

TAMs play a crucial role in the tumor microenvironment and have been extensively studied in various types of cancer, including gastric cancer (GC). Except for peripheral vasculature, molecules involved in signal transduction, the extracellular matrix (ECM), and tremendous cells without malignant functions are involved in the complex processes that make up the TME (66–68). TAMs are a type of immune cell that derives from monocytes and infiltrates the tumor microenvironment, where they can support tumor growth, angiogenesis, invasion, and immune suppression (69–71). The ECM is a key constitutive part of the TME, consisting principally of reactive tissue components including glycoproteins, collagens, and many enzymes that have an impact on cell adhesion, proliferation, and communication. These substances can change their physical properties, composition, and the environment in which they live to affect tumor cell migration, while the concentration of ECM also determines the ratio of tumor cell migration from one region to another (72–76). Additionally, nonmalignant cells in the TME are prominently separated into two primary groups, immune cells and mesenchymal stromal cells, which stimulate and promote uncontrolled cell proliferation to affect all stages of carcinogenesis (77–79). In the tumor microenvironment, immune cells are made up of both adaptive and intrinsic immune cells (80–82), and the two categories of lymphocytes, T and B lymphocytes, are essential integrants of adaptive immune cells. The two kinds of lymphocytes, T and B, are key ingredients of adaptive immune cells, while DC cells, NK cells, and macrophages are intrinsic cells involved in nonspecific immunity. Moreover, the signals delivered by the tumor and taking part in the responses and activities associated with tumor initiation and progression can influence these immune cells. Mesenchymal stromal cells, supportive cells recruited by cancerous tissues from the adjacent tissue mesenchyme, are vital to tumor formation. The composition of mesenchymal stromal cells varies among various kinds of tumors, and they involve endothelial cells, adipocytes, fibroblasts, and stellate cells, among which endothelial cells not only form neovessels to draw nutrients from the body for the tumor to grow but also, during tumor initiation and progression, endothelial cells assist tumor cells to escape from the immune system and prohibit them from harming the body’s immune system (83–85). Another valued cellular component is fibroblasts, which move tumor cells from the primary tumor site through the blood system to all parts of the body. Providing a reliable conduit for endothelial cells by fibroblasts can carry out angiogenesis (86–88). Adipocytes are specialized cells that regulate energy homeostasis in the body and can be involved in tumor progression through the secretion of metabolites, hormones, growth factors, and cytokines (89–91).

**Figure 2:**
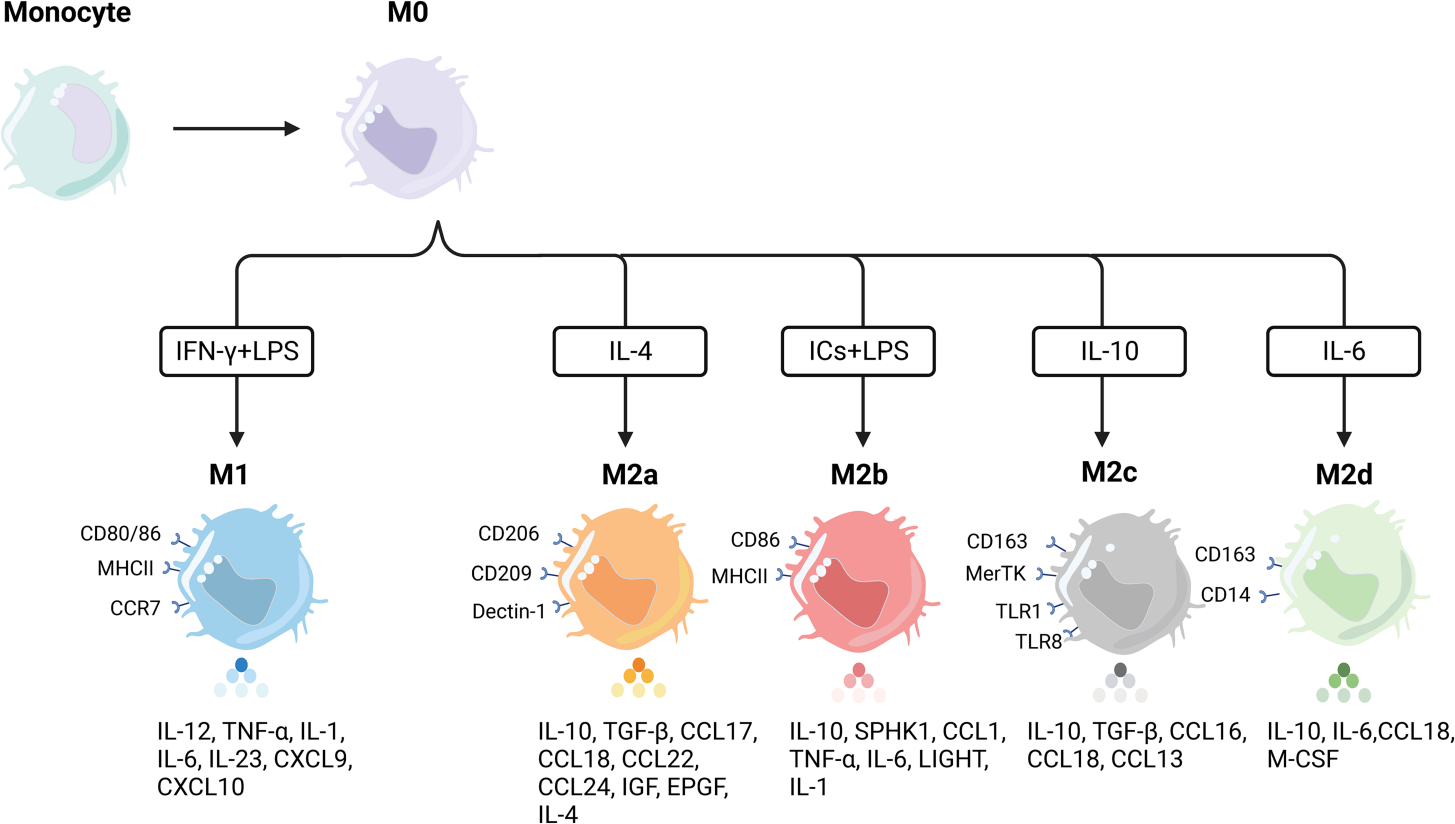
Polarization of macrophages and their characteristics. Macrophages undergo differentiation from precursor monocytes into undifferentiated M0 macrophages. In response to various stimuli, M0 macrophages can further differentiate into distinct subpopulations with specific phenotypic markers and cytokine secretion profiles. M1 macrophages, for instance, are typically induced by Th1 cytokines (e.g., IFN-γ) or bacterial lipopolysaccharides (LPS). This subset of macrophages displays elevated levels of proinflammatory cytokines, including TNF-α, IL-1α, IL-1β, IL-6, IL-12, and IL-23. On the other hand, M2 macrophages can be further divided into four distinct subpopulations based on the specific activation stimulus received. The induction of M2a macrophages involves IL-4, resulting in high levels of CD206 expression and increased secretion of TNF-β, IL-10, and IL-4, among others. M2b macrophages, in contrast, can be induced by both immune complexes (ICs) and LPS, leading to the production of anti-inflammatory and proinflammatory cytokines such as IL-10, TNF-α, and IL-6. M2 macrophages, induced by glucocorticoids and IL-10, exhibit strong anti-inflammatory effects on apoptotic cells through the release of high levels of IL-10 and transforming growth factor-beta. Lastly, M2d macrophages are induced by IL-6 alone and represent a distinct type of M2 macrophage.

Macrophages exist in almost every tissue and organ of the human body (92–94), originating predominantly from embryonic precursors before birth and from monocyte precursors of grown-up hematopoietic origin after birth. Macrophages, the staple part of the human phagocytic system, have a host of functions, taking part in physiological processes containing mammalian-specific and nonspecific defense (95, 96). Early research has detected that macrophages principally play a role in enhancing the progression of inflammation *in vivo* by phagocytosing bacteria, cellular debris, parasites, and senescent and abnormal cells, and by boosting the body’s self-healing process (97, 98). The proportion of macrophages in the total mass of cellular tumors in the TME is 15%–20%. They are the immune cells infiltrating the largest number of cells, enabling the manipulation and coordination of multiple elements intervening in tumor initiation, invasion, therapy resistance, and systemic metastasis (99–101). Some cytokines or signaling molecules in TME play a vital role in the polarization state and functional phenotype of macrophages (102). Lymphocytes, natural killer cells, and Th1 cells secrete IFN-γ to transform macrophages in the resting state into macrophages with antimicrobial and regulatory phagocytosis capacity, producing the immunostimulatory factors IL-12 and TNF-α to exert a proinflammatory progression effect, which is primarily manifested as inhibition of tumor progression in tumorigenesis, the M1-kind macrophages. Meanwhile, cytokines secreted by Th2 cells can activate resting macrophages, involving IL-4, IL-10, and IL-13, to differentiate into anti-inflammatory macrophages and produce diverse elements inhibiting inflammation, including IL-10, TGF-β, and Arg-1, which chiefly facilitate tumor progression during tumor initiation and progression, M2-type macrophages. M2-type macrophages, also known as TAMs, are abundant in the tumor microenvironment of cancer patients, with a predominance of M2-type, which plays an immunosuppressive role. The polarization process of macrophages and their characteristics are displayed in **Figure 2**. The number of TAMs infiltrated is associated with the tumor’s pathological stage and lymph node metastasis (103–105). In boosting tumor metastasis, M2-type macrophages can produce different sorts of enzymes in the form of paracrine secretion, which can arouse the destruction of collagen components involved in the composition of ECM and disintegrate the structure of ECM, which can benefit the migration of tumor cells (106, 107). Moreover, M2-type macrophages can release angiogenic molecules and express a string of enzymes involved in the regulation of angiogenesis to engage in tumor neovascularization, thereby further contributing to the malignant behavior of tumors (108–110).

## 4 Application of TAMs in GCs

In the early stages of GC, TAMs are recruited to the tumor site through chemokine signals and contribute to the initiation of GC. They are generally of an M2-like phenotype, which is associated with immunosuppression and tumor-promoting properties. TAMs secrete growth factors such as vascular endothelial growth factor (VEGF) and transforming growth factor-beta (TGF-β), promoting angiogenesis and tissue remodeling. As the progression of tumor invasion and metastasis, TAMs facilitate tumor invasion and metastasis through various mechanisms. They promote the epithelial-mesenchymal transition (EMT), a process that enables tumor cells to acquire a more invasive phenotype. TAM-derived factors, such as matrix metalloproteinases (MMPs), contribute to extracellular matrix degradation, allowing tumor cells to invade surrounding tissues and metastasize to distant sites. In the advanced/metastatic GC, TAMs can create an immunosuppressive microenvironment by inhibiting T-cell response, promoting regulatory T-cell (Tregs) accumulation, and producing anti-inflammatory cytokines like interleukin-10 (IL-10) and TGF-β. This immune suppression hinders anticancer immune responses and contributes to therapy resistance in the advanced stages of GC.

There is more research on exosomes, finding that exosomes are valued in the crosstalk between tumor cells and macrophages. Recent explorations have also verified that macrophages can stably take up tumor cell-derived exosomes and can be induced to shift toward an M2-polarized phenotype, thus affecting tumor malignant progression, therapeutic resistance, immunosuppression, and some other processes.

### 4.1 Tumor-derived exosomes modulate macrophage polarization

Rearrangement of cytoskeletal proteins activated by tumor-derived exosomes (TEX) is a primordial feature of macrophage activation and maturation, and the latter stimulates paracrine signaling pathways to enhance tumor growth infiltration, tumor-linked angiogenesis, tumor tissue inflammation, and immune remodeling. TEX influences the number and degree of macrophages that undergo polarization. While activated M1/M2-type macrophages function through their host tumor, the tumor’s immune microenvironment also plays a role, such as in tumor growth, migration, premetastatic niche (PMN) formation, and metastasis. The potential roles and mechanisms of tumor-derived exosomes in macrophage polarization are listed in **Table 1**. Ma et al. confirmed that ELFN1-AS1 was upregulated in GC tissues and cells and enriched in GC-derived exosomes. Exosomal ELFN1-AS1 improves cellular capacity, stemness maturity, metastasis, and M2 polarization of GC. The consequences of mechanistic experiments suggested that ELFN1-AS1 binds miR-4644 and boosts PKM expression. On top of that, exosomal ELFN1-AS1 can modulate PKM in a HIF-1α-dependent manner and promote M2 polarization and macrophage recruitment to regulate glycolysis in GCs (111). Xin et al. demonstrated that lncRNA HCG18 is enriched in GC-Exos and is favorable to M2 macrophage polarization. The outcomes of mechanistic investigations confirmed that lncRNA HCG18 could decline miR-875-3p in macrophages to strengthen the expression of KLF4 to inhibit the malignant progression of GCs and thereby help M2 macrophage polarization (112). Li et al. found that MIR4435-2HG expression was prominently boosted in GC and negatively related to the survival of GC patients, and that inhibition of MIR4435-2HG in cells decreased the viability and migration of GC cells. On top of that, exosomes deliver MIR4435-2HG, which induces macrophage M2 polarization by MKN45 cells, to macrophages. The outcomes of mechanistic studies demonstrated that MIR4435-2HG could benefit the Jagged1/Notch and JAK1/STAT3 pathways in macrophages to promote the malignant progression of GC (113). Song et al. detected that hsa_circ_0017252 was notably downregulated in GC tissues, which could sponge miR-17-5p to inhibit GC cell migration. Furthermore, hsa_circ_0017252 was enriched in GC cell exosomes and could be sent to macrophages to effectively inhibit macrophage M2-like polarization (114). Qiu et al. suggested that miR-519a-3p expression was markedly raised in the serum exosomes of patients with GC liver metastases (GC-LM) and that high expression of exosomal miR-519a-3p also predicted a poorer prognosis for individuals. Mechanistic tests confirmed that exo-miR-519a-3p could activate the MAPK/ERK pathway by targeting DUSP2, resulting in M2-like polarization of macrophages. M2-polarized macrophages further facilitate GC-LM formation by inducing angiogenesis and accelerating the formation of intrahepatic premetastatic niches (115).

**Table 1:** Potential roles and mechanisms of tumor-derived exosomes in macrophage polarization.

Molecular	Parent cell/source	Target cell	Target	Biological function	Reference
ELFN1-AS	GC cells	–	ELFN1-AS1/miR-4644/PKM	Enhance the cellular capacity, stemness, metastasis, and M2 polarization of GC, and regulate the glycolysis process	(111)
HCG18	GC cells	TAMs	HCG18/miR-875-3p/KLF4	Promote M2 macrophage polarization	(112)
MIR4435-2HG	GC cells	TAMs	MIR4435-2HG-Jagged1/Notch-JAK1/STAT3	Promote cell growth and migration and M2 macrophage polarization	(113)
hsa_circ_0017252	GC cells	TAMs	hsa_circ_0017252/miR-17-5p	Inhibit cell migration and M2 macrophage polarization	(114)
miR-519a-3p	GC cells	TAMs	miR-519a-3p/DUSP2/MAPK/ERK	Promote M2 macrophage polarization	(115)

Apart from ncRNAs, the regulatory effects of GC-derived exosomes on TAMs can also be affected after curing GC cells with drugs. Melatonin (MLT), a bioactive substance acting physiologically, was first separated and extracted from the bovine pineal gland (116, 117), which plays a valued role in the regulation of circadian rhythm, immunity, antioxidant capacity, and lipid metabolism in the organism (118, 119). Wang et al. detected that MLT treatment of GC cells changed the expression of microRNAs in cellular exosomes and regulated PD-L1 levels in macrophages, hence inhibiting their antitumor activity. Moreover, MLT also improved the secretion levels of TNF-α and CXCL10 in macrophages, and the exosomes secreted by MLT-treated GC cells promoted the recruitment of CD8^+^ T cells to the tumor site, hence inhibiting tumor growth (120). Wu et al. verified that modified Jianpi Yangzheng decoction (JPYZ) (mJPYZ) declined a great number of serum exosome PKM2 in advanced GC patients and xenograft tumor models. Furthermore, they are convinced that PKM2 is a packaging protein for GC cell exosomes, with mJPYZ reducing the delivery of tumor cell exosomal PKM2 to macrophages and alleviating exosomal PKM2-induced M2-TAM differentiation in the tumor microenvironment, consequently inhibiting GC progression. To conclude, macrophage-induced M2 differentiation can be promoted by PKM2-containing GC exosomes. Meanwhile, mJPYZ can inhibit the delivery of PKM2 to assist in the clinical treatment of GC (121).

**Figure 3:**
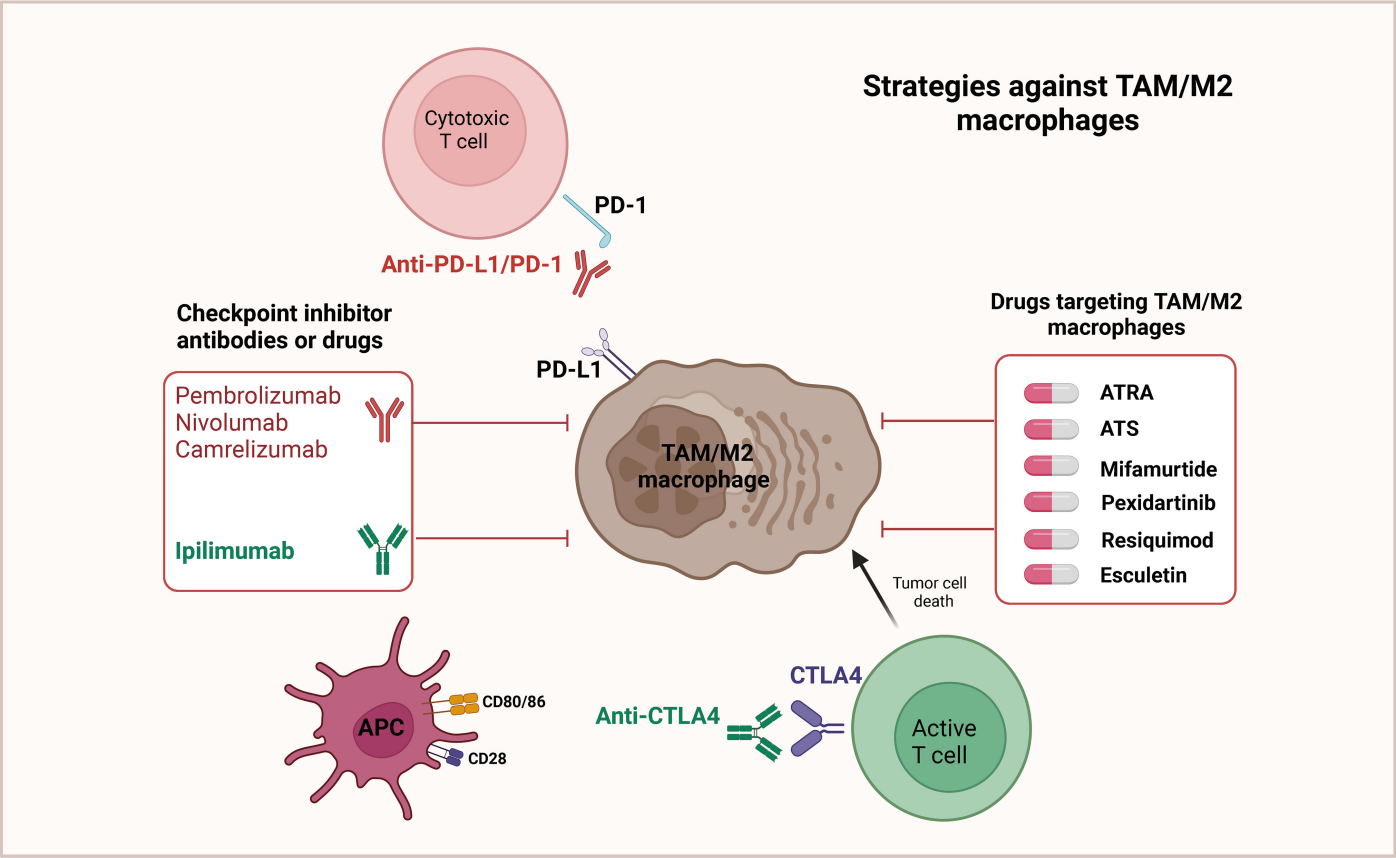
Targeted therapeutic strategies against TAM/M2 macrophages. In the tumor microenvironment, it has been observed that T cells display a notable level of PD-1 expression. This PD-1 molecule can interact with the ligand PD-L1, which is found on M2 macrophages as well as cancer cells. Such interactions have been found to result in the deactivation of T cells. Therefore, an effective strategy to restore the anticancer activity of T cells involves targeted inhibition of the PD-L1/PD-1 pathway. By suppressing this pathway, T cells can be reactivated, thereby reinstating their ability to combat cancer. Additionally, it has also been shown that repolarizing M2 macrophages to adopt the M1 phenotype can lead to a reduction in PD-L1 expression within the tumor microenvironment. Consequently, this further supports the development of various drugs that aim to repolarize TAMs/M2 macrophages.

PD-1 is an essential immunosuppressive molecule, a member of the CD28 superfamily, expressed on the surface of activated T and B lymphocytes (122–124). PD-L1 is expressed by tumor cells, bringing about the continuous activation of the PD-1/PD-L1 signaling pathway in the tumor microenvironment, in which negative feedback inhibits the activity of T/B lymphocytes, thereby mediating the occurrence of immune escape from tumor cells (125–127). It has been confirmed that the PD-1/PD-L1 signaling pathway is involved in the mechanism of the development of multiple solid tumors, and high expression of PD-L1 is relevant to poor prognosis. The targeted therapeutic strategies against TAM/M2 macrophages by regulating PD1/PD-L1 are shown in **Figure 3**. Wang et al. identified a macrophage primary subpopulation (PD1^+^ TAMs) constitutively expressing PD1 and aggregates in advanced GCs. These PD1^+^ TAMs exhibited M2-like surface features, in which the expression of CD206, IL-10, and CCL1 was notably increased, whereas the expression of MHC class II, CD64, and IL-12, as well as the phagocytosis of ovalbumin, were markedly decreased. Moreover, PD1^+^ TAMs triggered PD1 signaling, followed by inhibition of CD8^+^ T-cell function. Further experiments detected that GC-derived exosomes effectively induced PD1^+^ TAM production and generated a large number of IL-10, which impaired CD8^+^ T-cell function and contributed to the malignant progression of GC (128). Gu et al. verified that GC-derived exosomes (GC-exo) are numerous in the lungs and can be taken up by macrophages. Immunosuppressive phenotypic differentiation of macrophages in turn can be induced by them, which boosts the expression of PD-L1 through activation of the extracellular signal-regulated kinase (ERK) signaling pathway. High-throughput sequencing showed that miR-92a-3p was added to GC-exo and activated ERK signaling by inhibiting PTEN expression. Inhibition of ERK signaling with PD98059, a specific inhibitor, notably suppressed PD-L1 expression in macrophages and reversed the immunosuppressive impact of PMN, greatly inhibiting GC cell colonization in the lung (129).

### 4.2 Macrophage-derived exosomes are involved in GC progression

As a key constituent of the TME, macrophages frequently exhibit characteristics associated with an M2-like phenotype and collaborate with cancer cells to promote tumorigenesis, tumor progression, and resistance to therapy. The influence exerted by macrophages on the therapeutic response of cancer cells has garnered attention, leading researchers to explore their potential as targets for anticancer treatment. However, precise details regarding the intricate interactions between anticancer therapies and TAMs remain largely unknown.

#### 4.2.1 Regulating cancer proliferation, migration, and invasion

Experiments have shown that TAMs can manage the growth and metastasis of GC cells through exosomes and hereat encourage malignant tumor progression (**Table 2**). Zheng et al. proved that TAMs were enriched in GCs and could be converted to M2 polarization to foster the migration of GC cells. Otherwise, apolipoprotein E (ApoE) was also increased in M2 macrophages and could penetrate GC cells, activating the PI3K-Akt signaling pathway in recipient GC cells to remodel the cytoskeleton to support migration. To sum up, our findings indicate that exosome-mediated transfer of functional apolipoproteins from TAMs to tumor cells fosters GC cell migration (130). Zhang et al. proved that treatment of GC cells with TAM-exos notably promoted cell invasion and migration, while treatment with JPYZ markedly inhibited the expression of miR-513b-5p in TAM-exos. Mechanistic experiments indicated that miR-513b-5p could target and inhibit PTEN and then activate the AKT/mTOR signaling pathway, thus encouraging GC invasion and metastasis *in vivo* and *in vitro*. To conclude, TAM exosomes contain miR-513b-5p, leading to GC invasion and migration, and JPYZ may be an effective inhibitor (131). Yang et al. indicated that M2 macrophages encouraged GC cell proliferation while M1 macrophages did not, and using the exosome inhibitor GW4869 reduced the role of M2 macrophages. Also, miR-487a was increased in the exosomes of M2 macrophages. Delivering miR-487a to GC cells can induce cell proliferation and tumorigenesis. Mechanistic analysis implied that miR-487a fostered GC proliferation and tumorigenesis by targeting TIA1 (132).

**Table 2:** Potential roles and mechanisms of TAM-derived exosomes in cell proliferation and migration of GC.

Molecular	Parent cell/source	Target cell	Target	Biological function	Reference
ApoE	TAMs	GC cells	ApoE/PI3K-Akt	Promote GC metastasis	(130)
miR-513b-5p	TAMs	GC cells	miR-513b-5p/PTEN/AKT/mTOR	Promote GC invasion and metastasis	(131)
miR-487a	TAMs	GC cells	miR-487a/TIA1	Promote cell proliferation and tumorigenesis	(132)
miR-21	TAMs	GC cells	miR-21/PDCD4	Promote GC metastasis	(133)

Based on this, we can design targeted inhibitors to inhibit the malignant progression of GC and make clinical therapy come true. Previously, miR-21 could work as a pro-oncogenic factor in GC and foster tumor progression by targeting multiple downstream genes. Wang et al. detected that loading miR-21 inhibitors into macrophage-derived exosomes and delivering them to BGC-823 cells inhibited the migration ability of BGC-823 GC cells and encouraged apoptosis. Mechanistic investigations suggested that miR-21 could target the expression of PDCD4 to hold back apoptosis of GC cells, consequently boosting carcinogenesis. Briefly, the exosome-mediated transmission of miR-21 inhibitor possesses more functional inhibition and lower cytotoxicity with high efficiency (133).

### 4.3 Generating drug resistance

Drug resistance is one of the most important challenges in tumor therapy, which involves the resistance of tumor cells to the drug, consequently impairing the therapeutic effect and even causing treatment failure (134–136). This complex process includes multiple elements such as genetic mutations, epigenetic alterations, signaling pathway abnormalities, and the tumor microenvironment (137–140). The potential roles and mechanisms of TAM-derived exosomes in the drug resistance of GC are displayed in **Table 3**. Zheng et al. found that exosomes from M2 macrophages (M2-exos) facilitated DDP resistance in GC cells, and miR-21 grew in exosomes and cell lysates isolated from M2-polarized macrophages. Mechanistic experiments implied that macrophages could send miR-21 to GC cells through exosomes, which could downregulate the PTEN-activated PI3K/AKT signaling pathway to inhibit apoptosis, incurring the development of DDP resistance in GCs (145). Gao et al. discovered that both macrophage and macrophage-derived exosomes prominently encouraged doxorubicin resistance in GC cells, with miR-223 rising in macrophage-derived exosomes and being transmitted to GC cells. Inhibition of exosomal miR-223 expression in macrophages decreased the biological effects of exosomes on GC cells. Mechanistic experiments found that exosomal miR-223 boosted doxorubicin resistance in GC cells by inhibiting the expression of F-box and WD repeat domain-containing 7 (FBXW7). Also, miR-223 is enriched in the plasma exosomes of GC patients and is highly associated with doxorubicin resistance in GC patients (141). Cui et al. found that M2-polarized macrophages were considered to send exosomes of miR-588 to GC cells and strengthen the resistance of GC cells to DDP, and overexpression of miR-588 improved the growth of DDP-resistant GC cells. Mechanistic experiments indicated that miR-588 could encourage the proliferation and apoptosis of DDP-exposed GC cells by targeting CYLD. In summary, M2 macrophage exosome-derived miR-588 strengthens the resistance of GC cells to DDP by partially targeting CYLD (142). Xin et al. proved that the expression of LncRNA CRNDE was signally boosted in both cancer tissues and TAMs of GC patients, which was enriched in M2-exo and conveyed from M2 macrophages to GC cells by exosomes. Inhibition of CRNDE expression in M2-exo inhibited the proliferation and increase of CDDP-treated GCs. The outcomes of mechanistic experiments suggested that the ubiquitination of developmentally downregulated protein 4-1 (NEDD4-1)-mediated phosphatases and tensin homologs (PTEN) expressed was improved by CRNDE in neural precursor cells. Anyway, silencing CRNDE in M2-exo strengthened the sensitivity of GC cells to CDDP, whereas inhibition of PTEN expression weakened this sensitivity (143). Yu et al. confirmed that circ-0008253 is enriched in M2-Exos and can be conveyed from M2-Exos to GC cells. Overexpression of circ-0008253 signally improved cell viability, tumor size, and ABCG2 levels and weakened the sensitivity of GC cells to OXA (144).

**Table 3:** Potential roles and mechanisms of TAM-derived exosomes in drug resistance of GC.

Molecular	Parent cell/source	Target cell	Target	Biological function	Reference
miR-21	TAMs	GC cells	miR-2/PTEN/PI3K/AKT	Inhibit cell apoptosis and induce DDP resistance	(130)
miR-223	TAMs	GC cells	miR-223/FBXW7	Promote doxorubicin resistance	(141)
miR-588	TAMs	GC cells	miR-588/CYLD	Promote cell growth and induce DDP resistance	(142)
CRNDE	TAMs	GC cells	CRNDE/NEDD4-1/PTEN	Promote cell growth and induce CDDP resistance	(143)
circ-0008253	TAMs	GC cells	circ-0008253/ABCG2	Promote cell viability, and tumor size, and reduce sensitivity to OXA	(144)

#### 4.3.1 Promoting immune escape of tumor cells

Tumor immune escape is a process in which tumor cells evade the surveillance of the immune system, suppress the anti-tumor immune response, and enhance the proliferation, invasion, and metastasis of tumor cells (146–148). The elements (**Table 4**) affecting tumor immune escape are composed of the tumor cells themselves and the tumor-induced immunosuppressive microenvironment (156–158). Li et al. proved that miR-16-5p grew in M1 macrophage exosomes, was sent to GC cells, and inhibited tumor formation *in vitro* and *in vivo* by detecting PD-L1. Also, exosomal miR-16-5p triggers T-cell immune responses and thus inhibits GC progression (159). Wang et al. proved that M2-GC cells can absorb and internalize exos, promoting cell proliferation and migration and inhibiting apoptosis. The consequences of mechanistic experiments demonstrated that M2-Exos could improve the phosphorylation of P38 and the expression of programmed death ligand 1 (PD-L1) and therefore help GC progression, achieving immune escape through the increase in the expression of PD-L1 (160).

**Table 4:** Exosome-mediated immune escape of tumor cells.

	Secreted cells	Recipient cells	Target	Role and mechanism in immune suppression	Reference
Exosomal miRNAs					
miR-1246	Colon cancer	Macrophages	TGF-β signaling	miR-1246-expressing TAMs have enhanced TGF-β signaling, which increases the Treg population in mouse tumors and promotes immune suppression.	(149)
miR-23a-3p	Hepatocellular carcinoma	Macrophages	Akt-PDL1 pathway	miR-23a-3p inhibits PTEN and induces PDL1 expression, which decreases the CD8^+^ T-cell ratio and promotes T-cell apoptosis.	(150)
miR-208b	Colorectal cancer	T cells	Cell death factor 4 (PDCD4)	PDCD4 promotes CD4^+^ Treg expansion, which promotes CRC growth and oxaliplatin resistance.	(151)
miR-107	Gastric cancer	MDSC	DICER	miR-107 targets 3′UTRs of DICER and PTEN in MDSCs. DICER downregulation promotes MDSC expansion, whereas PTEN inhibition upregulates the PI3Kinase pathway and promotes proliferation.	(152)
Exosomal LncRNAs					
TUC339	Hepatocellular carcinoma	THP1 monocytes	IL-1β, TNF-α, CD86	TUC339 overexpression decreases production of proinflammatory cytokines IL-1β and TNF-α, T-cell activator CD86 expression, and phagocytic activity in THP1 cells.	(153)
RPPH1	Colorectal cancer	Macrophages	RPPH1	RPPH1 increases the expression of M2 macrophage markers CCL17, CCL18, CXCL8, IL-10, and TGF-β. M2-polarized macrophages promote CRC proliferation and metastasis.	(154)
CRNDE-h	Colorectal cancer	CD4^+^ T cells	RORγT	RORγT binds to IL-17 promotor and triggers CD4^+^ T-cell differentiation into immunosuppressive IL-17-producing Th17 cells.	(155)

## 5 Prospects and conclusion

The onset and progression of GC are complex and closely linked with environmental factors, genetic factors, and *H. pylori* infection (161, 162). Risk factors for the development of gastric cancer include men, advanced age, *H. pylori* infection, dietary structure, irregular eating, alcohol abuse, and genetic susceptibility, while increased intake of vegetables and fruits rich in vitamins and fiber can lessen the overall incidence ratio in the population (163, 164). Therapeutically, the cure of gastric cancer is primarily based on surgery combined with perioperative neoadjuvant chemotherapy, adjuvant chemotherapy, radiotherapy, targeted therapy, and other comprehensive means (165, 166). At that moment, the treatment of gastric cancer, containing immunotherapy, gene therapy, and targeted therapy, is widely being investigated, which is necessary to reveal the molecular mechanism of gastric cancer and identify more effective therapeutic targets and new biomarkers.

Exosomes serve as primary mediators of intercellular communication and possess the ability to alter the fate of both their own and other cells. Their impact on tumors is evident through their capacity to enhance tumor proliferation and drug resistance, initiate the formation of myofibroblasts, promote angiogenesis, facilitate the establishment of a premetastatic ecological niche, and induce immunosuppression. Extensive investigation has demonstrated the involvement of exosomes in numerous key biological processes, including angiogenesis, proliferation, invasion, and migration, as well as the recurrence of gastric cancer (GC). Furthermore, exosomes play a significant role in regulating drug resistance in GC cells, thereby contributing to the progression of GC. The presence of various biologically active substances within tumor cell exosomes renders them potential candidates for early diagnosis of GC. Moreover, their biocompatibility allows for their utilization as carriers of chemotherapeutic and immunotherapeutic drugs, facilitating drug delivery across biological barriers and improving drug targeting, thereby minimizing undesirable side effects. The promising capability of exosomes as carriers of drug-loaded cargo directed toward target cells underscores their vast potential for future applications.

As a chief infiltrating cell subpopulation in TME, the key role of macrophages in tumor progression has attracted great attention. TAM is an essential part of TME in diverse tumors and plays a significant role in the genesis, progression, angiogenesis, and immunosuppression of abundant cancers (167–170). Furthermore, TAM infiltration, connected with the prognosis of many tumors, can be taken advantage of as a prognostic indicator and a novel therapeutic target for many tumors (171–173). As a result, further comprehension of the interaction of TAM with tumor cells and the specific mechanism of immunosuppression in TME can help optimize the therapeutic regimen, and disrupting the malignant interactions between TAM and tumor cells can benefit and inhibit tumor progression. TAM is a vital part of the tumor microenvironment of GCs, influencing the malignant biological behaviors of GCs and playing an important role in the genesis and metastasis of GCs (174–176). In the tumor microenvironment, TAM produces a lot of inflammatory factors, growth factors, chemokines, and proteases through mutual crosstalk with GC cells and a variety of other cells, which play active roles in tumor growth, inhibition of apoptosis, angiogenesis, and lymphatic metastasis (177–179). Additionally, detecting the expression levels of these associated substances in GC patients helps judge the therapeutic effects and prognosis of GC. Thus, an in-depth understanding of the role of TAM-secreted related substances in GC is helpful in finding new ideas for the treatment of GC. As a prominent communication medium between cancer cells and immune cells, exosomes can contain changes in immune cells in tumor patients, and alterations in immune cell phenotype and function contribute to GC progression and immunosuppression.

Tumor progression can be accelerated by different cells in the tumor microenvironment through different immune escape mechanisms, like secretion of immunosuppressive factors, impaired antigen presentation, or apoptosis induction (180, 181). Exosomes play a dual role in GC progression, but the exact mechanism of action of exosome-stimulated immune effects is still unknown. As immune diagnosis and therapy progress in leaps and bounds, in the research relevant to GC, exosomes, and immunity, we should concentrate on the mechanism of exosomes acting on tumor cells and immune cells, comprehend how exosomes transport biomolecules to target cells and make immune influences, search for more precise immunotherapeutic targets, and provide a more solid and powerful experimental research basis for the clinic (182–184). Moreover, the worth of exosomes in the clinical diagnosis and treatment of GC, as well as in the prediction of recurrence and metastasis, needs to be further certified before translation to the clinic, with the target of offering a new strategy for the early diagnosis and individualized treatment of GC and a new hope for GC patients (185–187).

Briefly, this paper reviewed the interactions between GC cells and TAMs and the effects of alterations in the tumor microenvironment on GC, with an opinion to offer a theoretical basis and foundation for the clinical diagnosis and therapy of GC.

## Author contributions

YQ: Writing – original draft. GL: Writing – review & editing. NL: Writing – review & editing. YH: Writing – review & editing. HT: Writing – review & editing. CJ: Writing – original draft, Writing – review & editing.
